# Ear Infection and Its Associated Risk Factors in First Nations and Rural School-Aged Canadian Children

**DOI:** 10.1155/2016/1523897

**Published:** 2016-02-10

**Authors:** Chandima P. Karunanayake, William Albritton, Donna C. Rennie, Joshua A. Lawson, Laura McCallum, P. Jenny Gardipy, Jeremy Seeseequasis, Arnold Naytowhow, Louise Hagel, Kathleen McMullin, Vivian Ramsden, Sylvia Abonyi, Jo-Ann Episkenew, James A. Dosman, Punam Pahwa, The First Nations Lung Health Project Research Team, The Saskatchewan Rural Health Study Team

**Affiliations:** ^1^Canadian Centre for Health and Safety in Agriculture, University of Saskatchewan, 104 Clinic Place, Saskatoon, SK, Canada S7N 2Z4; ^2^Department of Microbiology & Immunology, University of Saskatchewan, Room 2D01, Health Sciences Building, 107 Wiggins Road, Saskatoon, SK, Canada S7N 5E5; ^3^College of Nursing, University of Saskatchewan, 104 Clinic Place, Saskatoon, SK, Canada S7N 2Z4; ^4^Department of Medicine, University of Saskatchewan, Royal University Hospital, 103 Hospital Drive, Saskatoon, SK, Canada S7N 0W8; ^5^Community A, SK, Canada; ^6^Community B, SK, Canada; ^7^Department of Academic Family Medicine, University of Saskatchewan, West Winds Primary Health Centre, 3311 Fairlight Drive, Saskatoon, SK, Canada S7M 3Y5; ^8^Department of Community Health & Epidemiology, College of Medicine, University of Saskatchewan, 107 Wiggins Road, Saskatoon, SK, Canada S7N 5E5; ^9^Indigenous Peoples' Health Research Centre, University of Regina, 3737 Wascana Parkway Regina, SK, Canada S4S 0A2

## Abstract

*Background.* Ear infections in children are a major health problem and may be associated with hearing impairment and delayed language development.* Objective.* To determine the prevalence and the associated risk factors of ear infections in children 6–17 years old residing on two reserves and rural areas in the province of Saskatchewan.* Methodology.* Data were provided from two rural cross-sectional children studies. Outcome variable of interest was presence/absence of an ear infection. Logistic regression analysis was conducted to examine the relationship between ear infection and the other covariates.* Results.* The prevalence of ear infection was 57.8% for rural Caucasian children and 43.6% for First Nations children living on-reserve. First Nations children had a lower risk of ear infection. Ear infection prevalence was positively associated with younger age; first born in the family; self-reported physician-diagnosed tonsillitis; self-reported physician-diagnosed asthma; and any respiratory related allergy. Protective effect of breastfeeding longer than three months was observed on the prevalence of ear infection.* Conclusions.* While ear infection is a prevalent condition of childhood, First Nations children were less likely to have a history of ear infections when compared to their rural Caucasian counterparts.

## 1. Introduction

Ear infections in children are a major health problem and may be associated with hearing impairment [1]; delayed speech and language development [2]; and academic and educational development [3]. The most commonly identified ear infection is known as acute otitis media which is caused by swelling and infection in the middle ear [4]. Chronic ear infections can affect a child's ability to learn and consequently may have a lifelong impact on his/her quality of life and overall development [5, 6]. It is estimated that almost all children will have had an ear infection by the age of five years. According to the Canadian National Longitudinal Survey of Children and Youth (NLSCY) in 2008/2009, 50% of the Canadian children aged 2 to 3 years have had at least one ear infection since birth and 13% of children had frequent (four or more) ear infections [7]. It was also reported that the rates of ear infection declined over the years 1994/1995 to 2008/2009 due to the reduction in exposure to environmental tobacco smoke [7]. It has been reported that approximately 69% of US children <12 years of age reported having at least one ear infection in their lifetime [8]. In general, ear infections are mild and resolve by themselves over a short period of time; however, if left untreated, this infection could lead to hearing loss, some point in the future [9]. Current recommendations for acute otitis media are to observe and treat with antibiotics only if the condition does not resolve in a few days, thus clinical assessment is important in managing the condition [10].

Research has shown that Aboriginal ethnicity [11–14] is an important risk factor for ear infections. These authors identified that there was a higher risk of having ear infections among Aboriginal children compared to non-Aboriginal children.

Other risk factors for ear infections include younger maternal age [14]; male sex [11, 12]; younger age [11, 12]; low birth weight [12]; low socioeconomic status [11, 12]; daycare attendance [11, 12, 15]; inadequate housing conditions [16]; lack of access to health care [16]; exposure to cigarette smoking [11, 12]; and mothers who smoke during pregnancy [14]. It was also noted that breastfed children were less likely to experience ear infection [6, 11–13, 16–18].

To our knowledge, there is no Canadian data available to compare prevalence rates associated with risk factors of ear infection in Aboriginal children or rural and urban Canadian school-aged children. However, we are currently conducting two cohort studies, one with rural children and the second with First Nations children living in two reserve communities in Saskatchewan. The objective of this paper was to determine the prevalence and associated risk factors for ear infection in children 6–17 years old living in rural areas of the province or residing on-reserve.

## 2. Materials and Methods

### 2.1. Terminology

The term “Aboriginal peoples” refers to descendants of the original inhabitants of North America. The Canadian constitution recognizes three groups of Aboriginal people: Indians (commonly referred to as First Nations who are registered Indians under the Indian Act of Canada), Métis, and Inuit. These are three separate peoples with unique heritages, languages, cultural practices, and spiritual beliefs [19, 20].

The rural population was defined as consisting of those persons living in towns and municipalities outside the commuting zone of larger urban centres with population of 10,000 or more [21].

### 2.2. Study Design and Population

Data obtained from the child components of baseline surveys from the Saskatchewan Rural Health Study (SRHS) [22, 23] and the First Nations Lung Health Project (FNLHP) [24] were pooled for this report. The brief description of the study design related to the child component for each study is given below.

#### 2.2.1. Study Design: SRHS Child Component

The overall description of the SRHS and details of the study designs for the adult and child components were described elsewhere [22, 23]. Briefly, schools that were located within the rural municipalities and small towns that participated in the adult phase were considered to be the target schools for the child component. In order to facilitate parents' and school authorities' understanding of potential concerns related to children's survey and assessments, the research team met the school district boards and principals. Forty-three schools within ten school divisions were identified. These ten school divisions were invited to participate and all agreed to participate. Of those forty-three schools identified, thirty-nine agreed to participate. Permission was sought to conduct cross-sectional surveys of children's health by parental report and following parental consent and child assent and clinical assessments (spirometry, allergy, and anthropomorphic testing) through the schools. This approach has been the methodological approach in other surveys of children's health by the investigators and has been shown to provide good response rates [25, 26]. Similar protocol was used for the FNLHP child component as well.

The study participants received a study package from the home room teacher to be taken home to their parents for completion. The package contained (i) the information letter; (ii) the baseline questionnaire to be completed by a parent or guardian; (iii) a consent form (parent or guardian); (iv) an assent form and information letter (child); and (v) a return envelope. Parents were asked to send the completed questionnaire, signed parental consent, and assent forms sealed in the return envelope to the home room teacher within two weeks. The completed or not returned sealed study packages were retrieved from the schools by the research team at the end of four weeks. A reminder letter was sent home two weeks after the initial contact. A total of 5667 study packages were distributed to students in Grades 1 to 12 in the study area schools and 2757 (49%) were returned, with 42% of the forms being completed (*n* = 2383). Of the 2,383 children, 301 were excluded from the final analysis due to missing data on key variables and identifying ethnicity other than Caucasians resulting in a final sample size of 2082 children. Of those 301, 147 were First Nations/Métis ancestry. Due to the unknown number of First Nations children in this group, they were not included in the analysis.

#### 2.2.2. Study Design: FNLHP Child Component

Overall description of the FNLHP and details of the study design for the adult component were described elsewhere [24]. Details of the child component were described in this section. Two reserve communities participated in the study. During the study period, children in Kindergarten (6 years old) to Grade 12 in Community A and Community B attended schools that were on-reserve (three schools) or a neighbouring school where many of the children from the reserve attended (one school) were eligible for participation. The potential study population was 603 First Nations children. In order to facilitate parents/caregivers and community understanding of potential concerns related to the children's survey and assessments, the research team met with the superintendent of the school divisions, the directors of education, persons holding the education portfolios at each of the reserves, and the school principals. In these four schools, permission to conduct the study was granted by different level of authorities.

A parents/caregivers' information session (supper night) was held at each school site prior to beginning the survey. An elder from each community was invited to attend the supper night who provided traditional knowledge and advice related to facilitating this project. During the gathering of information about the survey, consent and assent processes were discussed. Information about the survey, parent/caregiver and child involvement was provided through the school newsletters.

Prospective participants received a personally addressed study package from the classroom teacher to be taken home to their parents/caregivers. The package contained (i) cover letter and questionnaire to be completed by a parent or caregiver; (ii) parent-child information letter and consent/assent forms; and (iii) a return envelope. Parents or caregivers were asked to return the completed or uncompleted questionnaire, signed parental consent, and assent forms in a sealed envelope to the classroom teacher at the school within two weeks. For each survey returned, parents or caregivers received a $5 gift card. The completed, sealed study packages were retrieved from the schools by the research nurses after the two-week return date. A total of 603 study packages were distributed to students in Kindergarten (6 years old) to Grade 12 in the study area and 363 (60.2%) study packages were returned, with 58.2% of completed surveys (*n* = 351). Therefore, data from 351 First Nations children were used in the analysis.

Approval for the study protocols was obtained from the Biomedical Research Ethics Board at the University of Saskatchewan (# Bio: 10-177 for the SRHS and # Bio: 13-27 for the FNLHP) and permission to conduct the surveys was obtained from the directors of each school division in June, 2010, and from the school principals in December, 2010, for the SRHS, and in January, 2012, for the FNLHP.

### 2.3. Variables of Interest

In both studies, child study questionnaire included items describing sociodemographics, health status of the child, childhood diseases and infections, other illnesses, the lifestyle and home environment, health risk behaviours, access to health care factors, and family history of respiratory health.

Information was collected on the following variables.

*The Outcome Variable.* The outcome variable of interest was the presence/absence of an ear infection, based on the following question: “has a doctor ever said this child had an ear infection (Yes/No)?”; it is likely that reported ear infections include acute, chronic, and uncommon types. However, the nature or severity of ear infections was not reported.

*Individual Factors.* Information was collected on the following demographics: child's sex; child's age; first born child; and obesity. Obesity was derived according to age/sex specific classification cut-offs established by the International Obesity Task Force [27, 28], using parental report of weight and height. Information on the following individual variables expected to be associated with the ear infection was collected: asthma; tonsillitis; mother smoking during pregnancy; and whether or not this child was breastfed and the duration of breastfeeding.

*Contextual Factors.* Information was collected on the following contextual factors: difficulty accessing to health care in the past 12 months; presence/absence of smoke inside the house; crowding based on number of people living in the home; damage caused by dampness; mold/mildew signs; dampness; socioeconomic status based on the mother's education.

*Statistical Analysis.* Statistical analyses were conducted using SPSS version 22 (SPSS Inc., Armonk, NY: IBM Corp.). The comparison of two ethnic groups was conducted using Chi-square tests. Logistic regression models were used to predict the relationship between ear infection, outcome of interest (yes or no), and a set of explanatory variables. Based on bivariable analysis, variables with *p* < 0.20 became candidates for a multivariable model. The strength of associations was presented by adjusted odds ratios (OR_adj_) and their 95% confidence intervals (CI). All variables which were statistically significant (*p* < 0.05) as well as important covariates were retained in the final multivariable model. A parsimonious model was selected based on Hosmer-Lemeshow goodness-of-fit statistic [29].

## 3. Results

There were 2082 Caucasian children who participated in SRHS aged 6–17 years. There were 351 First Nations children who participated in the FNLHP aged 6–17 years. For this paper, a new variable, ethnicity, First Nations or Caucasian, was created. Hence, the study population contains data from 2433 children that was used for analysis. Mean age and standard deviation of the study population were 11.3 ± 3.3 years. The overall prevalence of ear infection was 55.8%.

Table 1 depicts the comparison of characteristics by ethnicity. Approximately, 57.8% of Caucasian children reported having ever been diagnosed with an ear infection compared to 43.6% First Nations children. Breastfeeding longer than three months (57.5% versus 40.1%, resp.), higher rates of maternal smoking during pregnancy (51.6% versus 19.9%, resp.), higher rates of exposure to passive smoke (43.9% versus 12.5%, resp.), and higher rates of obese children (12.8% versus 5.9%, resp.) were observed in the First Nations children compared with Caucasian children.

The univariate relationships between the environmental factors and personal factors or covariates and ear infection using unadjusted logistic regression are shown in Table 2. The First Nations children showed a lower prevalence of ear infection compared to Caucasian children. Younger children (6–11 yrs) were at higher risk of ever having been diagnosed with an ear infection compared to older children (12–17 yrs). First born children were more likely to have an ear infection compared to their younger siblings. Children breastfed longer than three months were protected against ear infection. Asthma, allergy, and tonsillitis were significant comorbid conditions for ear infection (Table 2).

Results of multivariate logistic regression analysis adjusted for covariates are presented in Table 3. The significant predictors of increased risk of ear infection were age, children in the age group of 6–11 years, tonsillitis, asthma and any respiratory related allergy, and being first born in the family, while there was decreased risk of ear infection associated with children who were breastfed longer than three months and ethnicity (First Nations versus Caucasians) (Table 3). We did not find associations between passive smoking exposure, low birth weight, and other environmental exposures with the report of ear infections.

## 4. Discussion

Otitis media (OM) or middle ear infection is a common disease among children under the age of 6 years. According to Kong and Coates [11], ear infections were common in two age groups: between 6 and 24 months of age and 4-5 years of age. This was due to weaning; exposure to environmental conditions; and attending daycare or Kindergarten. Most research conducted in this topic studied children aged under 5 years [13–15]. In this study, we were able to access the school-aged children aged 6–17 years from rural and First Nations communities. We assumed that the question “has a doctor ever said this child had an ear infection?” would capture all ear infection cases during their infancy to date. The results showed that the younger age group (6–11 years) had a higher risk of ear infections compared to the older age group. This could be due to the higher chance of younger children parents recalling the disease occurrence compared to older children.

Our results showed a greater prevalence of ear infection among Caucasian children compared with First Nations children (43.6% for First Nations/Métis children and 57.8% for Caucasian children). The association between ethnicity and ear infection has been reported previously [30]. In Australia, Indigenous children were at higher risk for earlier and more severe ear infections compared to non-Indigenous children [1, 11]. Thomson reported that the hospitalization rates for otitis media were six times more frequent for First Nations children than other children [31]. Findlay and Janz showed that, among children aged 0 to 3 years, 46% of First Nations children living off reserve had an ear infection compared to 40% of all Canadian children within the same age range [32]. Another Canadian study reported that Aboriginal status was one of the strongest (OR = 1.4) risk factors for otitis media in a population-based birth cohort [14]. The First Nations and Inuit Regional Health Survey (FNIRHS) conducted with reserve communities in all provinces of Canada in 1996/1997 revealed that the prevalence of ear infection was 15.8% for new born to 17 years old children and 20.3% for new born to five years old First Nations/Inuit children. On the other hand, the National Longitudinal Survey of Children and Youth (NLSCY) in 1994/1995 demonstrated that the prevalence of ear infection was 53% among newborn to three years old children excluding the children living on First Nations reserves [33]. The difference in prevalence rates could be due to several reasons: in the NLSCY, ear infection must have been diagnosed by a health professional, but, in FNIRHS, this was not the case [33]. Also, access to a primary care professional could vary by ethnicity. No data were available on whether First Nations parents wait longer before seeking care for otitis media compared to other parents [31]. Another important observation in the present study was higher prevalence of breastfeeding longer than three months among First Nations children compared with Caucasian children (57.5% versus 40.1%, resp.). This could be the reason for lower prevalence of ear infection observed among these First Nations children compared with Caucasian children in the current study. One earlier study also observed similar results. MacMillan et al. reported that, in children up to two years of age, 39% of First Nations/Inuit children compared with 24% of other Canadian children were breastfed for more than six months [33]. The results from 2006 Aboriginal Children's Survey reported that a higher percentage (over 72%) of First Nations/Métis children living off reserve were breastfed [32].

Many studies showed that breastfeeding has a protective effect on the development of otitis media [11, 30, 34]. This could be due to the immunological properties of breast milk, which contains specific antibodies against respiratory viruses. According to a recent study, not being breastfed at three months was a significant risk factor for ear infection [1]. Uhari et al. review reported that breastfeeding was strongly associated with a decreased risk for otitis media during the first year of life [35]. The period of breastfeeding had a significant impact on the occurrence of otitis media. Salah et al. [36] found that breastfeeding for less than three months was associated with an increased risk of developing otitis media compared with infants who were breastfed more than three months. Other authors have also found similar results [37, 38]. Similar to these studies, this study reported that children breastfed longer than three months were associated with a decreased risk of developing an ear infection compared with children who were not breastfed or breastfed for less than three months. This was statistically significant.

Studies have shown that ear infection develops significantly more often in boys than in girls [11, 30, 37]. The reason for sex dependence is unknown [11, 30]. This study did not report any significant difference in the prevalence of ear infection among boys and girls (56.2% versus 55.3%, resp.).

An early meta-analysis of risk factors for acute otitis media reported that having at least one sibling significantly increased the risk of having an ear infection [35]. Labout et al. [34] reported that having a sibling was associated with an increased risk of otitis media in the early life of a child. Other studies have reported siblings' history of acute otitis media was a strong predictor of acute otitis media [30, 37]. Another review of otitis media risk factors summarized that overcrowding in homes and families with a number of older siblings were risk factors [11]. Baraibar [30] reported that these positive relationships between ear infection and having siblings could be due to both environmental and genetic factors. Similar to these earlier studies, this study showed that first born children had a significantly higher prevalence of ear infections. In this study, we were unable to identify any association between overcrowding in homes and ear infection.

Recently, the relationship between otitis media and obesity has been discussed [39]. Two North American studies [40, 41] identified childhood obesity as an independent risk factor for ear infection. A significant relationship between childhood obesity and ear infection was not observed in this study although there was a greater trend towards ear infection if the child was obese compared with those who were not (61.3% versus 55.4%, resp.).

It is a well-known factor that exposure to cigarette smoke is a risk factor for the development of ear infection among children [7, 11, 12, 17, 30, 35, 42, 43]. Thomas [7] reported that the reduced exposure to tobacco smoke had contributed to the decreased prevalence of ear infections among young Canadian children during the period of 1994/1995 to 2008/2009. An Australian study reported that passive cigarette smoking was a risk factor for otitis media among both Aboriginal and non-Aboriginal children [44]. In addition, two studies found that parental smoking increased the risk of otitis media [1, 35] and one did not [34]. Similar to our study, Salah et al. did not find a significant association between exposure to passive smoke and ear infections [36]. In the present study, maternal smoking during pregnancy was not a significant risk factor for ear infection and a similar result was found by Eldeirawi and Persky [8]. In contrast, some studies reported maternal smoking during pregnancy as a risk factor for the development of ear infection [14, 45, 46].

In addition to the risk factors mentioned above, several comorbid conditions were associated with ear infection. There is limited research on the effect of allergy in the pathogenesis of otitis media in younger children [47]. Results of a meta-analysis revealed that the presence of allergy or atopy increased the risk of ear infection [43]. Hurst demonstrated that the current medical evidence supports the link between allergy and ear infection and that, for more than 85% of cases with chronic ear infection, allergy might be the contributing factor [48]. This study also reported similar associations with allergy and ear infection. In contrast, one other study showed no association [36].

Two studies looked at the association between ear infection and asthma [1, 8] and tonsillitis [1]. Both authors found that ear infection was statistically associated with asthma. Furthermore, a history of repeated ear infections was associated with increased prevalence of asthma in children [8]. The present study supported these findings and found significant associations between ear infections and asthma and tonsillitis. Other studies also reported upper respiratory tract infections as a comorbid condition for ear infection [30, 36, 43].

This study has several strengths and several limitations. Very few Canadian studies have examined the risk factors of ear infections among Caucasian and First Nations children together. This combined study surveyed a large number of children allowing robust results. This study included children from rural Saskatchewan and First Nations from two reserves in Saskatchewan. On the other hand, the two studies had moderate response rates (SRHS: 42.0%; FNLHP: 58.2%). First Nations children for this paper were coming from two sources and they were from two reserves in Saskatchewan (Community A: *n* = 195; 44.1% of ear infection prevalence and 54.9% of breastfeeding longer than three months; Community B: *n* = 156; 42.9% of ear infection prevalence and 60.9% of breastfeeding longer than three months). Therefore, there is a variation of prevalence rates of ear infection and breastfeeding across communities. Thus, we are unable to generalize these results to other First Nations communities.

In general, due to the cross-sectional nature of the study, one of the major limitations was the parent-reported survey recall-bias of disease history. No detailed information on income status and daycare attendance was available. Although we have obtained information of whether or not this child was breastfed and duration of breastfeeding, we have not collected the information on feeding practices (e.g., exclusive breastfeeding, formula, or both).

## 5. Conclusions

These results suggested that significant determinants of ear infection were younger age; self-reported physician-diagnosed tonsillitis and asthma; any respiratory allergy; first born; and ethnicity. Breastfeeding longer than three months was protective. While ear infection was a prevalent condition of childhood, children of First Nations heritage living in rural communities were less likely to have a history of ear infections when compared to their rural Caucasian counterparts.

## Figures and Tables

**Table 1:** Comparison of proportions among Caucasian and First Nations children.

Variables	Caucasians *n* = 2082 *n* (%)	First Nations *n* = 351 *n* (%)	*p* value^*∗*^
Ear infection	1204 (57.8)	153 (43.6)	**<0.0001**
Tonsillitis	624 (30.0)	67 (19.1)	**<0.0001**
Had operation to remove the tonsils	194 (9.3)	13 (3.7)	**0.001**
Doctor diagnosed asthma	314 (15.1)	61 (17.4)	0.270
Sex			
Male	1025 (49.2)	165 (47.0)	0.441
Female	1057 (50.8)	186 (53.0)	
Obese			
Yes	123 (5.9)	45 (12.8)	**<0.0001**
No	1959 (94.1)	306 (87.2)	
Mother's highest education			
<Grade 12	97 (4.7)	87 (24.8)	**<0.0001**
≥Grade 12	1985 (95.3)	264 (75.2)	
Child breastfed longer than three months	835 (40.1)	202 (57.5)	**<0.0001**
Mother smoked during pregnancy	414 (19.9)	181 (51.6)	**<0.0001**
First born	809 (38.9)	93 (26.5)	**<0.0001**
Exposure to passive smoking	260 (12.5)	154 (43.9)	**<0.0001**
Any respiratory allergy	608 (29.2)	71 (20.2)	**0.001**
Difficulty of accessing regular or on-going health care in past 12 months	152 (7.3)	17 (4.8)	0.094

^*∗*^Chi-squared test statistics was reported for comparisons.

**Table 2:** Bivariable logistic regression analysis of the association of physician diagnosed ear infection on personal and environmental factors (*n* = 2433).

	Ever diagnosed with ear infection		Unadjusted^*∗*^ odds ratio
	Yes/total	(%)	(95% CI)
Environmental factors			
Exposure to passive smoking			
Yes	226/414	54.6	0.94 (0.76, 1.17)
No	1131/2019	56.0	1.00
During past 12 months, water or dampness			
Yes	576/1015	56.7	1.07 (0.91, 1.26)
No	781/1418	55.1	1.00
House damage caused by dampness			
Yes	403/720	56.0	1.01 (0.85, 1.20)
No	954/1713	55.7	1.00
Signs of mold or mildew in home			
Yes	300/542	55.4	0.98 (0.81, 1.18)
No	1057/1891	55.9	1.00
Number of people in home			
≤4 people	718/1286	55.8	1.00 (0.86, 1.18)
>4 people	639/1147	55.7	1.00

Personal factors			
Age, in years			
6–11	766/1319	58.1	**1.23 (1.04, 1.44)**
12–17	591/1114	53.1	1.00
Sex			
Male	669/1190	56.2	1.04 (0.88, 1.22)
Female	688/1243	55.3	1.00
Obese			
Yes	103/168	61.3	1.28 (0.93, 1.76)
No	1254/2265	55.4	1.00
Ethnicity			
First Nations/Métis	153/351	43.6	**0.56 (0.45, 0.71)**
Caucasian	1204/2082	57.8	1.00
Mother's education			
<High school	90/184	48.9	**0.74 (0.55, 1.00)**
≥High school	1267/2249	56.3	1.00
Mother smoked during pregnancy			
Yes	330/595	55.5	0.98 (0.82, 1.18)
No	1027/1838	55.9	1.00
Breastfed longer than three months			
Yes	551/1037	53.1	**0.83 (0.71, 0.98)**
No	806/1396	57.7	1.00
First born			
Yes	540/902	59.9	**1.30 (1.10, 1.54)**
No	817/1531	53.4	1.00
Tonsillitis			
Yes	550/691	79.6	**4.52 (3.67, 5.56)**
No	807/1742	46.3	1.00
Asthma			
Yes	242/375	64.5	**1.53 (1.27, 1.83)**
No	1115/2058	54.2	1.00
Any respiratory related allergy			
Yes	429/679	63.2	**1.53 (1.27, 1.83)**
No	928/1754	52.9	1.00
Birth weight^#^			
Underweight (<2500 g)	89/143	62.2	1.25 (0.88, 1.78)
Overweight (>4000 g)	203/353	57.5	1.03 (0.82, 1.29)
Normal (≥2500 g to 4000 g)	1031/1814	56.8	1.00

^*∗*^Odds rations that are significantly different from 1.00 (*p* < 0.05) are in bold face. ^#^Missing values are present.

**Table 3:** Adjusted odds ratios (95% confidence intervals) based on multivariate logistic regression for associations with physician diagnosed ear infection.

Variable	OR_adj_ (95% CI)
Age, in years	
6–11	**1.48 (1.24, 1.76)**
12–17	1.00
Sex	
Male	1.00 (0.84, 1.19)
Female	1.00
Obese	
Yes	1.29 (0.92, 1.81)
No	1.00
Mother smoked during pregnancy	
Yes	1.12 (0.91, 1.38)
No	1.00
Tonsillitis	
Yes	**4.48 (3.62, 5.53)**
No	1.00
Any respiratory related allergy	
Yes	**1.27 (1.04, 1.56)**
No	1.00
Asthma	
Yes	**1.34 (1.04, 1.73)**
No	1.00
Ethnicity	
First Nations/Métis	**0.61 (0.48, 0.79)**
Caucasian	1.00
First born in the family	
Yes	**1.22 (1.02, 1.46)**
No	1.00
Breastfed longer than three months	
Yes	**0.84 (0.70, 1.00)**
No	1.00
